# Editorial: Genetics and epigenetics of fetal alcohol spectrum disorders

**DOI:** 10.3389/fgene.2015.00146

**Published:** 2015-04-16

**Authors:** Stephen Mason, Feng C. Zhou

**Affiliations:** ^1^Department of Anatomy and Cell Biology, Indiana University School of MedicineIndianapolis, IN, USA; ^2^Stark Neuroscience Research Institute, Indiana University School of MedicineIndianapolis, IN, USA; ^3^Indiana Alcohol Research Center, Indiana University School of MedicineIndianapolis, IN, USA; ^4^Department of Psychology, Indiana University-Purdue University at IndianapolisIndianapolis, IN, USA

**Keywords:** neurodevelopment, gene environment interactions, DNA methylation, neuroepigenetics, imprinting, transgenerational effects

Children born to mothers who drink during pregnancy are at risk for growth retardation, memory, learning, and cognitive deficits under a lifelong disability known as Fetal Alcohol Spectrum Disorders (FASD), which occurs at a high rate in the US (~1/100 live births) and worldwide. There are three outstanding features of FASD. (a) Humans have been associated with alcohol consumption dated as far back as 7000 BC (McGovern et al., 2004) and there is no sign of waning. (b) FASD can range across a large spectrum of severity, from the more severe Fetal Alcohol Syndrome (FAS) (encompassing facial, brain, and gross deformity) to the hard to detect, subtle mental dysfunctions. The Centers for Disease Control and Prevention (CDC) indicates that approximately 7–8% of pregnant women consume alcohol in US, but diagnosed FASD occurs in a much smaller percentage (CDC, 2012). There is strong evidence to indicate that genetic makeup is a major contributing factor to the differential vulnerability to FASD. (c) Alcohol's deleterious effect has recently been found to go beyond cellular toxicity, to affect epigenetics. The epigenetic chemical code, methylation and acetylation written on top of genomic base elements (e.g., DNA cytosine and histone tails), can confer 3D DNA packaging and fundamentally alter gene transcription. Alcohol has recently been recognized to have strong influences on methylation and acetylation (see Resendiz et al., 2014a) via alcohol metabolism. Furthermore, current evidence points to alcohol's influence on the interaction of genetic and epigenetic factors. These fascinating new views are the center of this eBook, which includes new data and an in-depth discussion of the recent findings and expert opinions. It is hoped that by elucidating the genetic x epigenetic (GxE) interaction at the center of fetal alcohol exposure, new insight will lead the community of scientists toward a greater understanding of this disease, and lay a foundation for prospective new treatments and interventions.

FASD is, theoretically, an avoidable disease. Unfortunately, there are many misconceptions and a lack of general public awareness of the profound causality of alcohol on developmental dysfunction. This eBook includes a public awareness effort with an *Opinion* from Singh et al. (2014), challenging the research community to convey the message that “at no time during pregnancy is alcohol 100% safe to drink.”

Is the differential vulnerability of mother and offspring to alcohol derived from the genetic difference rendered by the physiological environment (e.g., differential maternal placenta) or the fetal genotypes themselves? The forum begins with a study to tease out this entanglement. Gilliam (2014) analyzes this question by experimentally transferring the fertilized blastocysts of the alcohol sensitive C57BL/6N (B6) mice to that of alcohol resistant DBA/2 (D2) dams, and vice versa. The finding is intriguing, and the result is supported by another design directly exposing the embryos of B6 and D2 (with identical alcohol conditions) in an incubator thereby bypassing maternal influence (e.g., Ogawa et al., 2005; Chen et al., 2011) and further by *in vivo* study between lines of genetically diverse mice (see Loucks and Carvan, 2004; Anthony et al., 2010; Downing et al., 2012). These studies indicated that abnormal neurodevelopment depends not only on the dose and pattern of alcohol exposure but also on interactions among environmental, genetic, and maternal factors. Which genes underlie the abnormal development featured in FASD? The Smith et al. (2014) chapter has a succinct discussion of the multiple genomic factors that can increase vulnerability to facial deficits through pathways such as calcium-mediated neural crest apoptosis. Additionally, multiple genomic factors acting on different genetic backgrounds (e.g., in W98S vs. W98D chicks) may contribute to the differential sensitivity of the ethanol-imposed apoptosis. What differences lie within the genome that really impact the genetic contribution to alcohol vulnerability and resistance? To ultimately answer this question requires an interrogation of the entire genome at single nucleotide resolution. The paper “Genomic signatures in the differential vulnerability to fetal alcohol in B6 and D2 mice” did just that (Lossie et al., 2014). The single nucleotide variant (SNV) analysis captured ~900 genes on promoter regions where transcription factors bind (e.g., *Eya2, Csmd3*) that may affect transcription and on non-coding regions that may result in missense mutations leading to abnormal protein formation.

How does alcohol utilize GxE to turn neuroprogenitor cells away from their normal course? Goldowitz et al. (2014) used a B6XD2 mice crossing to demonstrate that alcohol exposure alters the γH2AX histone to mediate differential cerebral cortical apoptosis between genetic lines. It appears the neuroprogenitor cells used an intricate epigenetic program to guide neuronal differentiation and maturation. Resendiz et al. (2014b) present a timely and in-depth discussion of the normal neurodevelopmental epigenetic program and how alcohol deregulates this program. Alcohol exposure alters gene promoter methylation, histone modification, and deregulates non-coding RNA that challenges canonical gene expression and can result in an observable phenotype. Imprinted genes are known to play a particularly important role in human growth and development. Masemola et al. (2015) examined human blood cells to show that an FAS cohort in South Africa undergo CpG methylation changes in imprinting control regions (ICRs) (e.g., lower KvDMR1 and PEG3 DMR) that control allele specific gene expression. If such changes are also carried in the brain, they may contribute to neurodevelopmental abnormalities seen in FASD.

As discussed in Kleiber et al. (2014), genetics and epigenetics are intertwined and changes to this dynamic may last into adulthood, contributing to FASD (Lo and Zhou, 2014). These GxE interactions are also elucidated through examples presented in the previously introduced papers (Lossie et al., 2014; Smith et al., 2014). The complex interplay of genetic and epigenetic factors are also showcased in the altered hypothalamic-pituitary-axis axis, which may account for causality in mental impairment and cancer (Mead and Sarkar, 2014). Additionally, DNA methylation changes, if carried through the germ line, may affect multiple generations subsequent to alcohol exposure (Mead and Sarkar, 2014). Such complex transmission may be established through imprinting of parental genes, which defies Mendelian inheritance (Tunc-Ozcan et al., 2014). Further, validations of transgenerational effects of alcohol exposure are needed, and are in progress.

In summary, this eBook reveals that FASD has a genetic propensity perturbed by an environmental input which may in part be registered through epigenetics. Abnormal epigenetic marks may be accumulated over time or even generations. This emphasizes our original message that no specific level of drinking is ever safe during pregnancy. Finally, treatment and prevention of FASD would be best addressed by taking both genetic and epigenetic factors into consideration.

## Conflict of interest statement

The authors declare that the research was conducted in the absence of any commercial or financial relationships that could be construed as a potential conflict of interest.

## References

[B1] Anthony B. Vinci-Booher S. Wetherill L. Ward R. Goodlett C. Zhou F. C. (2010). Alcohol-induced facial dysmorphology in C57BL/6 mouse models of fetal alcohol spectrum disorder. Alcohol 44, 659–671. 10.1016/j.alcohol.2010.04.002 20570474 PMC2955190

[B2] CDC. (2012). Alcohol Use and Binge Drinking Among Women of Childbearing Age–United States, 2006–2010. MMWR Morbidity and Mortality Weekly Report. Available online at: http://www.cdc.gov/mmwr 22810267

[B3] Chen Y. Ozturk N. C. Ni L. Goodlett C. Zhou F. C. (2011). Strain differences in developmental vulnerability to alcohol exposure via embryo culture in mice. Alcohol. Clin. Exp. Res. 35, 1293–1304. 10.1111/j.1530-0277.2011.01465 21410487 PMC4445648

[B4] Downing C. Flink S. Florez-McClure M. L. Johnson T. E. Tabakoff B. Kechris K. J. (2012). Gene expression changes in C57BL/6J and DBA/2J mice following prenatal alcohol exposure. Alcohol. Clin. Exp. Res. 36, 1519–1529. 10.1111/j.1530-0277.2012.01757 22530671 PMC3407322

[B5] Gilliam D. (2014). Embryo transfers between C57BL/6J and DBA/2J mice: examination of a maternal effect on ethanol teratogenesis. Front. Genet. 5:436. 10.3389/fgene.2014.00436 25566321 PMC4263196

[B6] Goldowitz D. Lussier A. A. Boyle J. K. Wong K. Lattimer S. L. Dubose C.. (2014). Molecular pathways underpinning ethanol-induced neurodegeneration. Front. Genet. 5:203. 10.3389/fgene.2014.00203 25076964 PMC4097813

[B7] Kleiber M. L. Diehl E. J. Laufer B. I. Mantha K. Chokroborty-Hoque A. Alberry B.. (2014). Long-term genomic and epigenomic dysregulation as a consequence of prenatal alcohol exposure: a model for fetal alcohol spectrum disorders. Front. Genet. 5:161. 10.3389/fgene.2014.00161 24917881 PMC4040446

[B9] Lo C. L. Zhou F. C. (2014). Environmental alterations of epigenetics prior to the birth. Int. Rev. Neurobiol. 115, 1–49. 10.1016/B978-0-12-801311-3.00001-9 25131541 PMC4158947

[B10] Lossie A. C. Muir W. M. Lo C.-L. Timm F. Liu Y. Gray W.. (2014). Implications of genomic signatures in the differential vulnerability to fetal alcohol exposure in C57BL/6 and DBA/2 mice. Front. Genet. 5:173. 10.3389/fgene.2014.00173 24966868 PMC4052096

[B11] Loucks E. Carvan M. J. III. (2004). Strain-dependent effects of developmental ethanol exposure in zebrafish. Neurotoxicol. Teratol. 26, 745–755. 10.1016/j.ntt.2004.06.017 15451039

[B12] Masemola M. L. van der Merwe L. Lombard Z. Viljoen D. Ramsay M. (2015). Reduced DNA methylation at the PEG3 DMR and KvDMR1 loci in children exposed to alcohol in utero: a South African Fetal Alcohol Syndrome cohort study. Front. Genet. 6:85. 10.3389/fgene.2015.00085 25806045 PMC4354390

[B15] McGovern P. E. Zhang J. Tang J. Zhang Z. Hall G. R. Moreau R. A.. (2004). Fermented beverages of pre- and proto-historic China. Proc. Natl. Acad. Sci. U.S.A. 101, 17593–17598. 10.1073/pnas.0407921102 15590771 PMC539767

[B16] Mead E. A. Sarkar D. K. (2014). Fetal alcohol spectrum disorders and their transmission through genetic and epigenetic mechanisms. Front. Genet. 5:154. 10.3389/fgene.2014.00154 24917878 PMC4040491

[B18] Ogawa T. Kuwagata M. Ruiz J. Zhou F. C. (2005). Differential teratogenic effect of alcohol on embryonic development between C57BL/6 and DBA/2 mice: a new view. Alcohol. Clin. Exp. Res. 29, 855–863. 10.1097/01.ALC.0000163495.71181.10 15897731

[B19] Resendiz M. Lo C.-L. S. Badin J. K. Zhou F. C. (2014a). Alcohol metabolism and epigenetic methylation and acetylation, in Molecular Aspects of Alcohol and Nutrition, 1st Edn, ed Patel V. (Elsevier).

[B20] Resendiz M. Mason S. Lo C.-L. Zhou F. C. (2014b). Epigenetic regulation of the neural transcriptome and alcohol interference during development. Front. Genet. 5:285. 10.3389/fgene.2014.00285 25206361 PMC4144008

[B21] Singh S. M. Laufer B. I. Kapalanga J. (2014). Fetal alcohol and the right to be born healthy. Front. Genet. 5:356. 10.3389/fgene.2014.00356 25352863 PMC4195355

[B23] Smith S. M. Garic A. Berres M. E. Flentke G. R. (2014). Genomic factors that shape craniofacial outcome and neural crest vulnerability in FASD. Front. Genet. 5:224. 10.3389/fgene.2014.00224 25147554 PMC4124534

[B24] Tunc-Ozcan E. Sittig L. J. Harper K. M. Graf E. N. Redei E. E. (2014). Hypothesis: genetic and epigenetic risk factors interact to modulate vulnerability and resilience to FASD. Front. Genet. 5:261. 10.3389/fgene.2014.00261 25140173 PMC4122175

